# Systemic release of high mobility group box 1 (HMGB1) protein is associated with severe and fatal *Plasmodium falciparum* malaria

**DOI:** 10.1186/1475-2875-12-105

**Published:** 2013-03-19

**Authors:** Sarah J Higgins, Katharine Xing, Hani Kim, Dylan C Kain, Feng Wang, Aggrey Dhabangi, Charles Musoke, Christine M Cserti-Gazdewich, Kevin J Tracey, Kevin C Kain, W Conrad Liles

**Affiliations:** 1 Sandra A Rotman Laboratory, McLaughlin-Rotman Centre for Global Health, University Health Network/University of Toronto, Toronto, ON, Canada; 2 Department of Laboratory Medicine and Pathobiology, University of Toronto, Toronto, ON, Canada; 3 Makerere University College of Health Sciences, Kampala, Uganda; 4 Laboratory Medicine Program (Transfusion Medicine), University Health Network/University of Toronto, Toronto, ON, Canada; 5 Laboratory of Biomedical Science, Feinstein Institute for Medical Research, Manhasset, NY, USA; 6 Division of Infectious Diseases, Department of Medicine, University of Toronto, Toronto, ON, Canada; 7 Department of Medicine, University of Washington, 1959 Pacific Street, Box 354620, Seattle, WA, 98195-6420, USA

**Keywords:** Severe malaria, HMGB1, Biomarker, Pathogenesis, Inflammation

## Abstract

**Background:**

Severe falciparum malaria (SM) pathogenesis has been attributed, in part, to deleterious systemic host inflammatory responses to infection. High mobility group box 1 (HMGB1) protein is an important mediator of inflammation implicated in sepsis pathophysiology.

**Methods:**

Plasma levels of HMGB1 were quantified in a cohort of febrile Ugandan children with *Plasmodium falciparum* infection, enrolled in a prospective observational case-controlled study, using a commercial enzyme-linked immunosorbent assay. The utility of HMGB1 to distinguish severe malaria (SM; n = 70) from uncomplicated malaria (UM; n = 33) patients and fatal (n = 21) *versus* non-fatal (n = 82) malaria, at presentation, was examined. Receiver operating characteristic curve analysis was used to assess the prognostic accuracy of HMGB1. The ability of *P. falciparum*-parasitized erythrocytes to induce HMGB1 from peripheral blood mononuclear cells was assessed *in vitro*. The effect of an anti-HMGB1 neutralizing antibody on disease outcome was assessed in the experimental *Plasmodium berghei* ANKA rodent parasite model of SM. Mortality and parasitaemia was assessed daily and compared to isotype antibody-treated controls.

**Results:**

Elevated plasma HMGB1 levels at presentation were significantly associated with SM and a subsequent fatal outcome in paediatric patients with *P. falciparum* infection. *In vitro*, parasitized erythrocytes induced HMGB1 release from human peripheral blood mononuclear cells. Antibody-mediated neutralization of HMGB1 in the experimental murine model of severe malaria failed to reduce mortality.

**Conclusion:**

These data suggest that elevated HMGB1 is an informative prognostic marker of disease severity in human SM, but do not support HMGB1 as a viable target for therapeutic intervention in experimental murine SM.

## Background

*Plasmodium falciparum* malaria remains a leading cause of global morbidity and mortality [1]. Recent advances in malaria treatment and prevention, including first-line therapy with artemisinin-based drugs [2] and increased use of insecticide-treated bed nets [3] have the potential to reduce the global impact of malaria. However, severe malaria (SM) continues to be associated with a high risk of mortality and long-term morbidity in survivors. A detailed understanding of the key events mediating pathogenesis may facilitate improved management of SM, including the development of novel prognostic tools and therapeutic interventions. Although an appropriate immune response is important for parasite control and development of immunological memory for subsequent infection, an unbalanced or dysregulated inflammatory response to infection has been associated with deleterious clinical outcomes in malaria [4].

High mobility group box 1 (HMGB1) is a ubiquitous nuclear protein with recently described properties as a mediator of inflammation [5]. Release of HMGB1 into the extracellular milieu, by either active secretion from immune effector cells and/or passive release from dying cells, acts as a ‘danger signal’ to trigger inflammation via interaction with pattern recognition receptors (PRR), including receptor for advanced glycation end product (RAGE) and toll-like receptor (TLR) family members, resulting in increased pro-inflammatory cytokine gene transcription and production [5].

Studies in experimental models of sepsis, a life threatening syndrome of systemic inflammation with pathophysiological features resembling SM (including vascular permeability, and multi-organ dysfunction) [4], suggest that HMGB1 is involved in mediating sepsis-related pathology [5]. Preclinical studies have reported that neutralizing anti-HMGB1 antibodies prevents organ damage and lethality in established models of experimental sepsis (both endotoxin induced- and cecal ligation and puncture (CLP)-induced models) [6-8], even with late administration [7]. These studies suggest that HMGB1 may represent a novel therapeutic target to reduce deleterious inflammation during systemic infection [5].

The potential role of HMGB1 in the pathogenesis of infectious syndromes associated with pronounced systemic inflammation suggests that HMGB1 may contribute to disease severity and outcome in malaria. This study examined HMGB1 release during *P. falciparum* infection and the association of HMGB1 release with disease severity and mortality. The potential therapeutic efficacy of HMGB1 neutralization was investigated in a murine model of SM using an anti-HMGB1 monoclonal antibody (mAb) with previously validated therapeutic benefit in experimental sepsis models.

## Methods

### Study population and ethics statement

Febrile pediatric patients (ages 6 months to 12 years) with microscopy-confirmed *P. falciparum* infection were eligible for enrollment in a prospective observational nested case-controlled study conducted at Mulago Hospital’s Acute Care Unit in Kampala, Uganda between October 15, 2007 and October 30, 2009, as described [9,10]. Exclusion criteria included any of the following: severe malnutrition, HIV co-infection, known previous enrolment in the study, absence of adequate consent or absence of any laboratory specimens.

Upon enrollment, clinical and demographic data and venous blood samples were collected. Citrate plasma derived from venous blood samples was aliquoted and stored at −80°C until testing. Thin and thick blood smears obtained at presentation were reviewed at a reference parasitology laboratory by two independent experts to determine parasite density using leucocyte counts and confirm malaria diagnosis. Samples for biomarker testing were derived from the larger study cohort based on availability of sufficient volume of unthawed plasma samples. Patients who fulfilled World Health Organization (WHO) sub-categorization of malaria syndromes, including cerebral malaria (CM), severe malaria anaemia (SMA) and/or respiratory distress with either hypoxia or lactic acidosis [11], and were under inpatient treatment, were categorized as severe malaria (SM). Patients not fulfilling this criteria and under treatment as outpatients were defined as having uncomplicated malaria (UM) and enrolled as age-matched controls.

Ethical approval for the study was obtained from the Mulago Hospital Research Ethics Committee, Makerere University Faculty of Medicine Research Ethics Committee, Uganda National Council for Science & Technology, and Toronto Academic Health Sciences Network Research Ethics Board (University Health Network). Written informed consent was obtained from the parents or guardians of all participants.

### Measurement of human plasma HMGB1 levels by ELISA

Plasma HMGB1 levels were quantified using a commercial enzyme-linked immunosorbent assay (ELISA) kit, according to the manufacturer’s instruction (Shino-Test Corporation).

### Peripheral blood mononuclear cell (PBMC)-*Plasmodium falciparum* co-culture system

Human PBMCs were isolated from malaria naïve volunteers using Ficoll-Paque (GE Healthcare). Cells were cultured at 1.5 × 10^6^ cells/well in RPMI 1640 medium supplemented with 10% FBS and 2.5% gentamycin (Invitrogen) in the presence of *P. falciparum* (confirmed *Mycoplasma*-free ITG strain)-infected erythrocytes (PEs; 4.5 × 10^6^/well), uninfected erythrocytes (uEs; 4.5 × 10^6^/well), lipopolysaccharide (LPS; 100 ng/ml), or RPMI 1640 alone for two, six, or 24 hours at 37°C and 5% CO2. At each of the time point, supernatant was collected and concentrated 10-fold with Vivaspin 10000 MWCO centrifugal concentrators at 15,000 g, 10 min, room temperature.

### Measurement of HMGB1 in cultured supernatants by Western blot

Cultured supernatants were concentrated 10-fold with Vivaspin 10000MWCO centrifugal concentrators (15, 000 g), resolved by 10% SDS-PAGE and transferred onto a PVDF membrane for immunoblotting. Blots were probed with with a rabbit polyclonal anti-HMGB1 antibody (1:2,000; Abcam) and secondary HRP-conjugated anti-rabbit antibody (1:3000). Protein bands were visualized using SuperSignal West Pico Chemiluminescent substrate (Pierce).

### Murine experimental severe malaria model

Infection in eight- to 10-week old female C57BL/6 mice (Jackson Laboratories) was initiated by intraperitoneal (ip) injection of 1 × 10^6^ freshly isolated *Plasmodium berghei* ANKA (MR4)-PEs. Parasitaemia was monitored by thin-blood smear stained with modified Giemsa (Sigma). Mice were monitored twice daily for signs of experimental severe/cerebral malaria (paralysis, ataxia, convulsions and/or coma). Mice judged as developing severe/cerebral malaria were euthanized by CO_2_. Experiments mice were performed in accordance with the University Health Network Animal Care Committee guidelines and regulations.

For antibody administration, animals were randomized to receive either anti-HMGB1 monoclonal antibody (2G7, mouse IgG2b; 50 ug/mouse), isotype (non-immune IgG2b; 50 ug/mouse) or vehicle (PBS) control via ip injection every other day beginning one day prior to PbA infection until day 5 (d5) post PbA infection (pi).

### Measurement of mouse plasma HMGB1, TNF, IFNγ, IL-6, IL-10, MCP-1 and IL-12 levels

Peripheral blood was collected by either saphenous venipuncture on day 0 (d0; prior to PbA infection) and d5 post pi in heparinized tubes (Starstedt) or, for time course analysis experiments, blood was collected at specified time points by cardiac puncture into heparinized syringe following euthanasia by CO_2_. Blood was centrifuged and plasma was collected and stored at −80°C. Plasma cytokine (TNF, IL-6, IL-10, IL-12 and IFNγ) and chemokine (MCP-1) levels were measured using the mouse inflammation cytometric bead array kit (CBA; BD Biosciences), according to the manufacturer’s protocol. Plasma HMGB1 levels were quantified (i) using a commercial enzyme-linked immunosorbent assay (ELISA) kit, according to the manufacturer’s instruction (Shino-Test Corporation) and (ii) by western blot analysis, as follows. Plasma samples were diluted in cell lysis buffer (Cell Signaling Technology), mixed with an equivalent volume of the 2× Laemmli buffer containing 100 mM dithiothreitol, and boiled for 5 min. Plasma samples were separated by SDS-PAGE, and transferred onto PVDF membrane for immunoblotting. Blots were probed with rabbit HMGB-1 monoclonal antibody (clone EPR3507, 1:1000, Abcam). For quantification, blots were scanned and band densities determined by using the NIH Image-J software.

### Statistical analysis

Statistical analysis was performed using Prism v4 (GraphPad). Differences between groups were assessed using a non-parametric Mann–Whitney or Fischer test, as appropriate, except for the comparison of HMGB1 expression in plasma collected from the same mouse at different time points, which was measured by the paired *t*-test. Multiple groups were compared using Kruskal-Wallis test with Dunn’s multiple comparison test. Receiver operating characteristic (ROC) curves and area under the curve (AUC) were generated to assess the predictive accuracy. To correct for multiple comparisons, p values were adjusted using Bonferroni-Holm’s correction. ECM survival studies were compared by Log-rank test and visualized by generation of Kaplan-Meier plots. Statistical significance was defined as a p value of less than 0.05, unless otherwise specified.

## Results

### Systemic release of high mobility group box 1 (HMGB1) protein is associated with severe and fatal *Plasmodium falciparum* malaria

**Figure 1:**
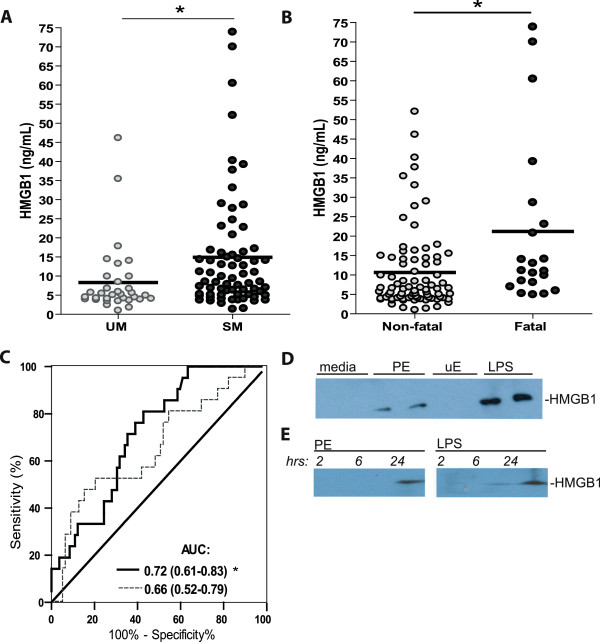
**HMGB1 is released with *Plasmodium falciparum *infection and predictive of malarial disease severity and clinical outcome in Ugandan children.** Plasma HMGB1 levels at clinical presentation are significantly higher in children with severe malaria (SM) compared to uncomplicated malaria (UM) (**A**) and in fatal cases when compared to non-fatal cases (**B**). Statistical analysis by Mann Whitney *U* test. * indicates a p value of <0.01. (**C**) Receiver operating characteristics (ROC) curves were generated for HMGB1 (solid line) and parasitaemia (dashed line) to assess the utility in predicting outcome. Area under the curve (AUC) is displayed with 95% confidence intervals in parentheses. p values were adjusted for multiple comparisons using Bonferroni-Holm’s correction (n = 2). * indicates a significant p value of <0.025 (**D**) HMGB1 is detectable in cultured media 24 hours following peripheral blood mononuclear cells (PBMC; 1.5 × 10^6^ per well) (isolated from healthy, malaria-naïve donors) stimulation with parasitized erythrocytes PEs (4.5 × 10^6^ per well) or LPS (100 ng/ml) but not after stimulation with uninfected erythrocytes (uE; 4.5 × 10^6^ per well) or media alone. HMGB1 was analysed by western blot using rabbit polyclonal anti-HMGB1 antibodies. (**E**) The kinetics of HMGB1 release from PBMCs (1.5 × 10^6^ per well) was assessed two, six and 24 hours after stimulated with PEs (4.5 × 10^6^ per well) or LPS (100 ng/ml).

In this study, extracellular HMGB1 was quantified in plasma from 103 febrile Ugandan children with either uncomplicated malaria (UM) or SM (Table 1) by ELISA [9]. Admission levels of HMGB1 were significantly higher in children with smear-confirmed severe *P. falciparum* malaria compared to UM (Figure 1A). Furthermore, HMGB1 levels at admission were significantly elevated in children who subsequently died from *P. falciparum* malaria compared to non-fatal cases (Figure 1B). Receiver operating characteristics (ROC) curves generated to assess the ability of HMGB1 to discriminate between non-fatal *versus* fatal *P. falciparum* malaria showed HMGB1 had an acceptable predictive utility (AUC = 0.72 p = 0.002; Figure 1C). Notably, HMGB1 performed better than the peripheral blood parasite count (AUC = 0.66 p = *ns* after correction for multiple comparisons).

**Table 1:** **Demographic and clinical data for study population **^**$**^

**Characteristic**	**UM(n = 33)**	**SM(n = 70)**	**p value**	**Pooled samples**		**p value**
				**Non-fatal(n = 82)**	**Fatal(n = 21)**	
**Age (years)**	3.0 (1.7-6.75)	1.9 (1.0-3.3)	***0.0065***	2.25 (1.2-4.4)	2.05 (1.2-3.55)	0.86
**Weight (kg)**	13.0 (10.0 -21.0)	10.5 (8.5-13.0)	***0.0044***	11.0 (9.0-15.0)	11.5 (10.0-13.0)	0.71
**Female (%)**	14 (42.4)	37 (52.1)	0.4	37 (45.1)	14 (63.6)	0.15
**Days unwell prior to presentation**	3 (2–4)	3 (3–4)	0.32	3 (3–4)	3.5 (2–6.5)	0.87
**Parasitaemia (parasites/uL)**	3.9 × 10^4^ (6.1 × 10^3^-1.4 × 10^5^)	4.0 × 10^4^ (9.2 × 10^3^-1.7 × 10^5^)	0.59	3.8 × 10^4^ (6.3 × 10^3^-1.4 × 10^5^)	1.2 × 10^5^ (1.4 × 10^3^-3.6 × 10^5^)	0.074

UM = uncomplicated malaria, SM = severe malaria (includes cerebral malaria (CM; n = 32), severe malarial anaemia (SMA; n = 35), CM/SMA (n = 1), non-CM/SMA (n = 3)).

^$^All variables are presented as median (interquartile range) unless otherwise indicated. Groups were compared using Mann Whitney U or Fisher exact test.

Western blot analysis of cultured media showed that HMGB1 was released by PBMCs exposed to *P. falciparum-*PEs *in vitro* (Figure 1D), with later kinetics compared to lipopolysaccharide-induced HMGB1 release (Figure 1D and E).

### Systemic levels of HMGB1 are increased during *Plasmodium berghei* ANKA infection in mice susceptible to development of experimental severe/cerebral malaria (ECM)

**Figure 2:**
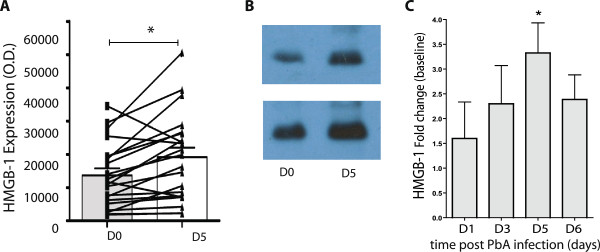
**HMGB1 is elevated in plasma of mice susceptible to developing severe and fatal malaria following *Plasmodium berghei *ANKA infection.** Plasma expression levels of HMGB1 were measured by Western blot from plasma collected on day 5 post-infection with *Plasmodium berghei*-ANKA, a rodent adapted parasite strain (**A-B**) Band intensities for D0 (grey bar) and D5 (white bar) in each group were compared on the same blot by using the NIH Image J software. Statistically significant differences between D0 and D5 were assessed by the paired *t*-test in each group. * indicates a p value of <0.05 (p = 0.004). Histograms represent mean + SEM obtained from a pooled result from at least two independent Western blot analyses from two independent PbA infection studies (n = 15-20/group). Representative Western blots are shown in panel (**B**). Systemic levels of HMGB1 in plasma over the course of PbA infection (on day 1, 3, 5 and 6 post infection) were assessed by ELISA in ECM-susceptible C57BL/6 mice (**C**). HMGB1 levels are shown as the fold change compared to the geometric mean of baseline HMGB1 levels obtained from naïve, uninfected littermate C57BL/6 mice. Bars represent mean + SEM (n = 5/time point). Statistical significant difference between day 1 and day 5 levels were determined by Kruskal Wallis with Dunn’s multiple comparison test. * indicates a p value of <0.05.

To investigate a potential causal role for HMGB1 in severe malaria, the PbA model of experimental severe/cerebral malaria (ECM) was utilized. In this model, ECM-susceptible C57BL/6 mice develop clinical signs of severe illness approximately five to six days after PbA infection. HMGB1 levels in ECM-susceptible mice were determined by Western blot and ELISA. For Western blot analysis, quantitative comparisons of the HMGB1 band densities were made only for bands blotted on the same membrane (Figure 2A and B). All blots were repeated at least once to account for blot-to-blot variability, using plasma samples collected from at least two independent infection studies. Basal levels of HMGB1 were highly variable between mice, therefore comparisons were made between baseline levels for the same mouse, when possible, or the fold induction compared to average baseline levels of uninfected naïve littermate mice. Exposure to PbA-PEs resulted in incremental elevation in circulating extracellular plasma HMGB1 levels (expressed as fold change compared to baseline) (Figure 2C), with significant elevation by d5 post-infection compared to respective basal levels (p < 0.05), Figure 2A to C), demonstrating HMGB1 release in response to malaria infection, similar to observations in human clinical malaria and cultured PBMCs exposed to *P. falciparum*-PEs (Figure 1A, B, D and E).

### Treatment using a HMGB1 neutralizing mAb does not affect disease outcome, parasitaemia or cytokine levels in ECM-susceptible mice

**Figure 3:**
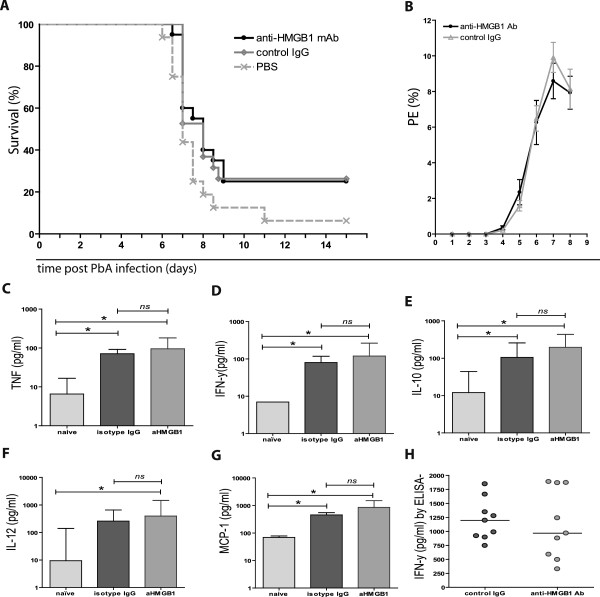
**HMGB1 is not necessary or sufficient for the development of severe malaria following *Plasmodium berghei *ANKA (PbA) infection in the experimental murine model.** (**A**) Kaplan-Meier survival analysis after C57BL/6 mice were infected with 1 × 10^6^*Plasmodium berghei*-ANKA (PbA) infected erythrocytes. There was no significant difference in survival between mice receiving an HMGB1 neutralizing antibody (2G7) (black circle, solid line), or control IgG2b (grey diamond, solid line) (Log rank test, p = 0.9, median survival = eight days for both groups; n = 20/group) or PBS control (grey x, dashed line) (Log rank test, p = 0.06; n = 15). Data is pooled from two independent experiments. The kinetics of peripheral parasitaemia are shown up to day 9 post PbA infection for anti-HMGB1 2G7 treatment mice and isotype IgG control (**B**). Results expressed as a percentage of the parasitized erythrocytes (PE) and represent mean + SEM from one representative experiment (n = 10/group). Levels of TNF, IFN-γ, IL-10, IL-12 and MCP-1 were measured in serum collected from naïve, uninfected mice and PbA-infected mice on day 5 pi using the mouse cytometric bead array (**C**-**G**). Bars and whisker represent median and interquartile range (IQR). Statistical differences were assessed by Kruskal-Wallis one-way analysis of variance with Dunn’s multiple comparisons test. * indicates a p value of <0.05. Plasma levels of IFN-γ were confirmed by ELISA (**H**) and statistical difference between anti-HMGB1 2G7 and isotype IgG control was assessed by Mann–Whitney test (*ns*, p = 0.73). The line represents the median levels of all individual values in the scatter dot plot.

Based on the hypothesis that HMGB1 released during infection contributes to disease pathogenesis, the therapeutic efficacy of an HMGB1 neutralizing monoclonal antibody (2G7) was investigated in ECM-susceptible mice, at a dose previously shown to confer protection in experimental sepsis models [8]. Treatment with mAb 2G7 was not sufficient to improve outcome following PbA infection in susceptible mice. Both 2G7-treated and isotype control-treated mice developed PbA-associated ECM and succumbed to infection, with a mean survival time of eight days (Figure 3A). No difference in peripheral parasitaemia was observed between mice receiving 2G7 and isotope control (Figure 3B).

Because the immune response to infection is recognized as an important mediator in the development of ECM pathogenesis [12-16] and anti-HMGB1 2G7 mAb treatment has been shown to reduce levels of cytokine mediators released from HMGB1-stimulated macrophages *in vitro*[17] and reduce serum levels of cytokines induced by CLP in an experimental sepsis model [8], the ability of 2G7 to modulate excessive inflammation associated with PbA-infection was assessed. As expected, levels of TNF, IL-10, IL-12 and IFN-γ were significantly increased in all experimental PbA-infected mice at d5 pi compared to naïve, uninfected mice (Figure 3C to G). Plasma IL-6 levels were below the limit of detection for the assay and therefore were not included. Anti-HMBG1 mAb (2G7) treatment did not significantly alter circulating plasma levels of pro-inflammatory cytokines (TNF, IL-12 and IFN-γ), the anti-inflammatory cytokine IL-10 or the chemokine MCP-1 (Figure 3C to G). IFN-y levels were further confirmed by ELISA analysis using plasma samples collected from an independent infection study. As observed with CBA analysis, anti-HMGB1 mAb treatment used in this study did not affect IFN-γ release induced during PbA infection, as levels were comparable to plasma IFN-γ levels in PbA-infected isotype controls (Figure 3H).

## Discussion

Prognostic and diagnostic biomarkers of underlying pathological processes may represent promising tools to supplement clinical and laboratory assessment and improve triage and clinical management of SM. In this study, HMGB1 levels at clinical presentation were increased in a cohort of Ugandan children with severe *P. falciparum* malaria, confirming and extending previous reports of elevated extracellular HMGB1 in fatal paediatric falciparum cases [18]. Moreover, HMGB1 levels were predictive of malarial disease severity and clinical outcome, suggesting that quantification of extracellular HMGB1 may be a useful prognostic marker of severe and fatal malaria. HMGB1 performed better as a prognostic indicator than the peripheral blood parasite count, a parameter commonly used as a prognostic indicator in falciparum malaria. The predictive accuracy of HMGB1 was comparable to other acute phase biomarkers associated with inflammatory conditions, such as procalcitonin (PCT) (AUC = 0.72) and soluble triggering receptor expressed on myeloid cells-1 (sTREM-1) (AUC = 0.76), previously reported to improve clinical performance (sensitivity >90% and specificity >80%) when used in biomarker combinations to predict mortality in children with severe malaria [19]. These results indicate that HMGB1 may represent a novel host-derived biomarker that may contribute unique information and further improve predicative accuracy when integrated into combinatorial biomarker panels.

Extracellular HMGB1 is believed to act as a danger signal and initiates a host immune response resulting in increased pro-inflammatory production. Using a peripheral blood mononuclear cell (PBMC)-*P. falciparum* co-culture approach, *P. falciparum*-PEs were shown in this study to induce HMGB1 release from human PBMCs, which may account for elevated plasma/serum levels observed in malaria patients. It was hypothesized that malaria-induced release of HMGB1 from immune effector cells could be involved in the propagation of inflammation leading to malaria-associated immunopathology. If so, HMGB1-based strategies might represent a novel therapeutic approach for severe *P. falciparum* infection, as proposed for sepsis [5]. In the *P. berghei* ANKA murine model, the development of ECM is highly dependent on host genetics and immune response to infection. Mice lacking key inflammatory mediators, such as IFN-γ and members of the TNF superfamily (e g, LTα), are protected against the development of ECM [12-16]. Specific strategies to modulate the host immune response in this model have been reported to decrease disease severity and improve survival [12].

In this study, HMGB1 release was modulated by *Plasmodium* infection and increased in the peripheral blood of ECM-susceptible mice following infection, similar to observation in human populations, suggesting a potential role for HMGB1 in disease progression. However, administration of a monoclonal anti-HMGB1 antibody (2G7), given prophylactically at a dose previously shown to confer protection in experimental sepsis models [8], did not improve survival or modulate peripheral levels of key inflammatory markers. This study suggests that, unlike sepsis models, HMGB1-based interventions directed at the specific epitope targeted by anti-HMGB1 2G7 are not likely to be efficacious in the prevention of experimental SM in PbA-infected C57BL/6 mice.

Further studies are required to explain the failure of anti-HMGB-1 antibody-based intervention in this model. In the current study, anti-HMGB1 treatment did not affect circulating levels of inflammatory cytokines induced by PbA infection, although the same antibody has been shown to attenuate levels of inflammatory cytokines induced by CLP in an experimental sepsis model [8]. This could suggest that excessive pro-inflammatory responses in this model are not mediated by HMGB1, as has been previously described for other inflammatory diseases. Some, but not all, studies suggest that HMGB1 does not have direct cytokine activity but instead functions as a complex with TLR ligands (e g, LPS) to enhance or promote their effects, a function that may not be relevant for severe malaria pathology caused by malaria toxins or by-products. TLR4 has been identified as a principal receptor that meditates HMGB1-induced cytokine production and immunopathology [5]. Although the exact role for TLRs in CM remains to be elucidated, a number of studies suggest that the pathogenesis of PbA-induced SM is independent of TLR4 [20], unlike the pathogenesis of sepsis where mice deficient in TLR4 are highly resistant to the development of LPS-induced septic shock [21]. Although the study does not support the use of this anti-HMGB1 mAb, at the dose employed, as treatment in this context, it does not rule out a role for extracellular HMGB1 in the pathogenesis of *P. falciparum*-induced CM in humans. Further studies to elucidate the role of HMGB1, using strategies not employed and/or outside the scope of the current study, are warranted. It is also important to note that this study was carried out with a single neutralizing monoclonal antibody. It is possible that additional strategies to block HMGB1, including antibodies raised against the B box subunit domain of HMBG1, may yield more favourable outcomes.

Accumulating evidence indicates that the host response to infection contributes to the pathogenesis of SM. Improved understanding of the pathophysiological mechanisms of SM may lead to novel prognostic tools and therapeutic strategies to improve clinical outcome. In this study, HMGB1 levels at presentation were correlated with falciparum malaria disease severity in a cohort of paediatric patients, and there was a significant difference in admission HMGB1 levels between children who subsequently died from their infection *versus* those who survived. This study supports further investigation into the potential use of HMGB1 as a biomarker to assess disease severity and prognosis in paediatric malaria. Additional prospective, multicentre studies of SM in areas of varying malaria transmission are required to validate the clinical utility of HMGB1. However, based on the results of this study in a mouse model of SM, HMGB1 neutralization using anti-HMGB1 2G7 mAb does not appear to be a viable therapeutic strategy to improve clinical outcome in this model of severe malaria. Further studies are warranted to elucidate the role of HMGB1 in the pathogenesis of human and experimental SM and CM.

## Competing interests

The authors declare that they have no competing interests.

## Authors’ contributions

SJH performed experiments, statistical analysis and drafted the manuscript. KX performed the immunoassay. HK and DCK participated in the murine studies. FW performed the *in vitro* studies. AD, CM and CMC-G contributed to the study design and collection of patient samples. KJT provided reagents. KCK and WCL were involved in the conception and design of the study and drafting the manuscript. All authors read and approved the final manuscript.
